# Differences in Gut Microbiota between Healthy Individuals and Patients with Perianal Abscess before and after Surgery

**DOI:** 10.1155/2023/1165916

**Published:** 2023-04-12

**Authors:** Hezhai Yin, Bairu Luo, Qi Wang, Zhonghua Hong, Huilin Chen, Lidong Shen, Bin Shen, Bo Hu

**Affiliations:** ^1^Department of Surgery, Jiaxing Hospital of Traditional Chinese Medicine, Zhejiang Chinese Medical University, Jiaxing, 314001 Zhejiang, China; ^2^Department of Clinical Pathology, Jiaxing University Master Degree Cultivation Base, Jiaxing Hospital of Traditional Chinese Medicine, Zhejiang Chinese Medical University, Jiaxing, 314001 Zhejiang, China

## Abstract

Surgery is the most important treatment for perianal abscesses. However, the gut microbiota of patients with perianal abscess and the effects of perianal abscess on the gut microbiota after surgery are unknown. In this study, significant changes in interleukin 6 and tumor necrosis factor-*α* in the blood of healthy subjects, patients with perianal abscesses, and patients after perianal abscess surgery were identified. 16S rRNA gene sequencing technology was used to detect the changes in the gut microbiota among 30 healthy individuals and 30 patients with perianal abscess before and after surgery. Venn diagrams and alpha diversity analyses indicated differences in the abundance and uniformity of gut microbiota between the healthy individuals and patients with perianal abscesses before and after surgery. Beta diversity analysis indicated that the grouping effects among the control, abscess, and surgery groups were good. The classification and compositional analysis showed significant differences in the gut microbiota between healthy individuals and patients with perianal abscesses before and after surgery. LEfSe analysis, random forest analysis, and ROC curve analysis showed that *Klebsiella* (AUC = 0.7467) and *Bilophila* (AUC = 0.72) could be potential biomarkers for the diagnosis of perianal abscess. The functional prediction results showed that the differential microbiota is significantly enriched in the pathways related to nutrition and drug metabolism. This study may have important implications for the clinical management and prognostic assessment of patients with perianal abscesses.

## 1. Introduction

Perianal abscess is the most common type of anorectal abscess, causing severe discomfort to the patients [1, 2]. It occurs at the edge of the anus and, if left untreated, can extend to the ischial anal space or sphincter space because these areas are in continuation with the perianal space and can also cause systemic infections [1, 3]. Pain, swelling, and fever are considered to be their hallmark symptoms, and about 35% of the patients develop an anal fistula [4, 5]. By exploring the records of hospital inpatients and outpatients, it was found that the perianal abscesses and anal fistulas are increasing every year in various countries [6]. Especially the men aged 21-40 years are more likely to develop this disease [7]. The most common treatment for perianal abscesses is incision and drainage [8, 9]. Generally, the patients with perianal abscesses do not need follow-up treatments after surgery, but the pathogenic microorganisms can easily colonize the intestinal tract of patients after surgery [10]. Therefore, the changes in the gut microbiota of the patients with perianal abscesses after surgery are also needed to be focused on. Although perianal abscesses are regarded as nonthreatening conditions, they might affect the well-being and quality of patients' lives and the expensive conditions of society [11]. Therefore, clinicians must find appropriate therapeutic interventions for perianal abscesses.

The gut microbiota plays a huge role in some of the physiological processes in human body, such as biosynthesis of vitamins and amino acids, decomposition of food components, resistance against pathogenic microorganisms, prevention of epithelial damage, development and training of immune system, promotion of angiogenesis, storage of fats, and modification of nervous and immune actions [12–15]. The studies on human microbiota have demonstrated not only their physiological but also the pathological effects of gut microbiota on human health. The imbalance of gut microbiota can cause a series of diseases, including digestive system diseases, metabolic diseases, inflammatory bowel disease, irritable bowel syndrome, and autoimmune diseases [16–18]. Anus is the gateway of digestive tract to the outer body and the perianal diseases are closely related to the microorganisms [19]. At present, the correlations between the perianal abscesses and gut microbiota are not fully understood. Although studying the aerobic and anaerobic organisms, such as *Bacteroides fragilis*, *Peptostreptococcus*, *Prevotella*, *Fusobacterium*, *Porphyromonas*, *Clostridium* species, *Staphylococcus aureus*, *Streptococcus*, and *Escherichia coli*, are considered to be related to the perianal abscesses, their occurrence has a certain connection with gut microbiota [10]. Although uncomplicated perianal fistula can be treated by drainage, abscess formation usually requires emergency surgery [20, 21]. In addition, since many patients use anti-inflammatory or immunosuppressive drugs, additional antibiotic treatment is usually included to obtain the best treatment [22]. Although the use of antibiotics has been proved to have a positive effect on discharged anorectal abscess and is recommended, it is very important to adjust the types of antibiotics according to the microbial spectrum to avoid overtreatment and antibiotic resistance [20]. In addition, antibiotic treatment should not be delayed, and the individual characteristics of patients, such as complications and drug treatment, as well as potential microbial spectrum should be considered [22]. This is especially important for patients with perianal fistula and abscess formation because they often receive immunosuppressive or anti-inflammatory drugs. However, at present, there is little evidence about the formation of microbial spectrum of perianal abscess, and empirical antibiotic treatment is often insufficient [23]. Although some studies have detected that microorganisms, such as gram-negative bacilli and gram-positive cocci, such as *Escherichia coli* or *Streptococcus*, are generally related to the formation of anorectal abscess, there is no data on the microbial spectrum of patients with perianal abscess [22]. Therefore, it is particularly important to study the differences of bacterial flora in patients with perianal abscess before and after surgery and before and after drug treatment.

In this study, a high-throughput *16S rRNA* gene sequencing technology was used to identify the composition of gut microbiota in the fecal samples of healthy individuals and patients with perianal abscesses before and after surgery. The high-throughput *16S rRNA* gene sequencing technology could effectively detect the differences between the gut microbiota of healthy individuals and patients with perianal abscesses before and after surgery. This study is of great significance for the patients with perianal abscesses to select sensitive antibiotics for preventing the microbial colonization of possible pathogenic bacteria, as well as for their clinical treatment and prognostic evaluation.

## 2. Material and Methods

### 2.1. Research Design

The patients newly diagnosed (June-August, 2022) with perianal abscesses by a gastroenterologist were registered for this study. Inclusion criteria for the patients included diagnosis of perianal abscess, agreement to join the microbiota research project, and understanding of the research purpose. Exclusion criteria for the patients included diagnoses of other digestive diseases, such as colorectal cancer and diarrhea, severe cardiovascular diseases, pregnancy, pregnancy or lactation preparation, infectious diseases, local skin trauma or infections, diabetes, hypertension, and lack of informed consent. The feces of 30 healthy people and 30 patients with perianal abscess were collected for subsequent experiments. According to the diagnostic criteria of Gaertner et al., clinical blood tests and contact are used to identify patients with perianal abscess [24]. Collection of feces for follow-up experiments in patients with perianal abscess was done one week after surgery. Healthy people, patients with perianal abscess, and patients after perianal abscess surgery were the control group, abscess group, and surgical group, respectively. The study was approved by the Jiaxing Hospital of Traditional Chinese Medicine, and the participants signed a written informed consent before participating (ChiCTR2200061815).

### 2.2. Sample Collection

Stool specimens from all the participants were collected at the time of registration into 10 mL sterile centrifuge tubes immediately after defecation. The stool samples were frozen within 4 hours and stored at -80°C. All the frozen samples were processed within 6 months and shipped to LC-Bio Technologies (Hangzhou) Co., Ltd., for *16S rRNA* gene sequencing. Meanwhile, blood samples were collected from three groups of patients for subsequent testing of inflammatory factors.

### 2.3. Blood Test for Interleukin 6 (IL-6) and Tumor Necrosis Factor-*α* (TNF-*α*)

Blood samples were centrifuged at 3000 r/min, and serum was separated for the subsequent detection of inflammatory factors. The Roche Chemiluminescence e602 (Roche, Switzerland) was used for the detection of IL-6 and TNF-*α* in blood samples according to the reagent manufacturer's instructions (IMMULITE1000, Siemens, Germany).

### 2.4. DNA Extraction

Following the manufacturer's instructions, E.Z.N.A. ®Stool DNA Kit (D4015, Omega, Inc., USA) was used for the total bacterial DNA extraction from the stool samples [25]. Nuclease-free water was used as a negative control. The extracted DNA was eluted in a 50 *μ*L elution buffer and stored at -80°C until amplification using PCR by LC-Bio Technologies (Hangzhou) Co., Ltd., Hangzhou, Zhejiang Province, China.

### 2.5. PCR Amplification and 16S rRNA Gene Sequencing

The bacterial V3-V4 hypervariable regions of *16S rRNA* gene were amplified with primers 341F (5′-CCTACGGGNGGCWGCAG-3′) and 805R (5′-GACTACHVGGGTATCTAATCC-3′) [25]. The 5′ ends of the primers were tagged with specific barcodes per sample and sequenced using universal primers. The PCR amplification was performed on a 25 *μ*L reaction mixture, containing 25 ng of template DNA, 12.5 *μ*L of PCR Premix, 2.5 *μ*L of each primer, and PCR-grade water to adjust the total volume to 25 *μ*L. The conditions for PCR amplification were as follows: initial denaturation at 98°C for 30 seconds; 32 cycles of denaturation at 98°C for 10 seconds, annealing at 54°C for 30 seconds, extension at 72°C for 45 seconds, and final extension at 72°C for 10 minutes. The PCR products were confirmed on 2% agarose gel. Ultrapure water was used instead of template DNA as a negative control to exclude the possibility of false-positive PCR results. The PCR products were purified using AMPure XT beads (Beckman Coulter Genomics, Danvers, MA, USA) and quantified using Qubit (Invitrogen, USA). The amplicon libraries were prepared for sequencing. The size and quantity of the amplicon libraries were assessed using Agilent 2100 Bioanalyzer (Agilent, USA) and Library Quantification Kit for Illumina (Kapa Biosciences, Woburn, MA, USA), respectively. The libraries were sequenced on an Illumina NovaSeq PE250 platform.

### 2.6. Data Analysis

The samples were sequenced on an Illumina NovaSeq platform following the manufacturer's instructions provided by LC-Bio Technologies (Hangzhou) Co., Ltd. Paired-end sequencing reads were assigned to the samples based on their unique barcodes and truncated by cutting off the barcodes and primer sequences. The paired-end sequencing reads were merged using the FLASH software. The quality filtration of the raw sequencing reads was performed under specific filtering conditions to obtain the high-quality clean sequence reads according to the fqtrim (v0.94). Chimeric sequences were filtered using the VSEARCH software (v2.3.4). After removing duplicated sequences using DADA2, the feature table and feature sequences were obtained. The alpha and beta diversities were calculated by normalizing to the same sequences randomly. Then, according to SILVA database (release 132) classifier, the feature abundance was normalized using the relative abundance of each sample. The alpha diversity was applied to analyze the complexity of species diversity for a sample using 4 indices, including Chao1, observed species, Shannon, and Simpson indices; all these indices were calculated using QIIME2 (v2019.4). The beta diversity was also calculated using QIIME2 (v2019.4), and the graphs were drawn using R package. BLAST was used for the sequence alignment, and the feature sequences were annotated using the SILVA database for each of the representative sequences. LEfSe (linear discriminant analysis effect size) was performed to detect differentially abundant taxa across groups using the default parameters, and species with significant multigroup differences was detected by using the Kruskal-Wallis test. Other diagrams were implemented using the R package (v3.5.2).

## 3. Results

### 3.1. Study Subjects

**Figure 1:**
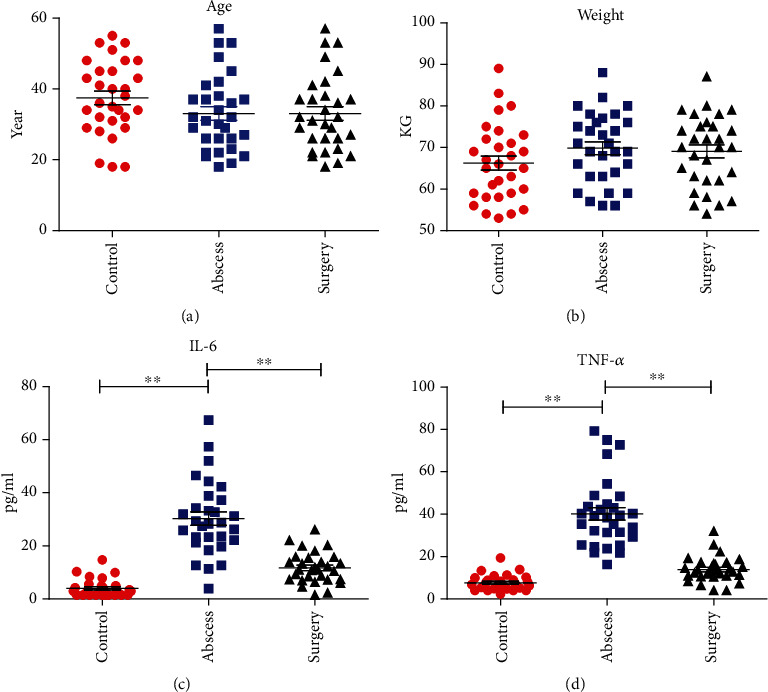
(a) Ages of the 30 healthy individuals and 30 patients with perianal abscess. (b) Weight of the 30 healthy individuals and 30 patients with perianal abscess. (c) Detection of IL-6 in blood samples. (d) Detection of TNF-*α* in blood samples. Wilcoxon test was used to test the significance of the differences between the three groups. ^∗^*p* < 0.05, ^∗∗^*p* < 0.01.

A total of 30 male subjects, including 30 healthy individuals and 30 patients with perianal abscesses, were included in this study. The ages of the subjects were within the range of 19 and 60 years, which are shown in Figure 1(a). As shown in Figure 1(b), there was no significant difference in body weight between patients in the control group, abscess group, and surgical group (*p* > 0.05). IL-6 and TNF-*α* were significantly higher in patients in the abscess group compared with the control group (*p* < 0.01). In addition, IL-6 and TNF-*α* were significantly decreased in patients in the surgical group compared with the abscess group (*p* < 0.01). This indicates that an inflammatory response occurred in patients with perianal abscess and that the inflammatory response in postoperative patients was suppressed by treatment.

### 3.2. Difference of *α*-Diversities of Gut Microbiota before and after Surgery in the Healthy Individuals and Patients with Perianal Abscess

**Figure 2:**
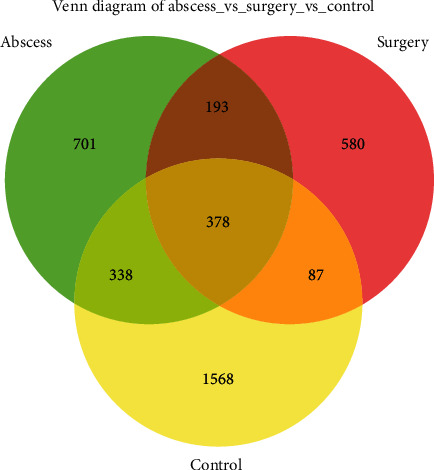
Venn diagram of the composition of ASVs in gut microbiota.

A total of 4,010,545 amplicon sequence variants (ASVs) were obtained after filtration using QIIME2 (v2019.4) with DADA2. The Venn diagram shows the shared and unique ASVs among the control, abscess, and surgery groups (Figure 2). There were 378 ASVs shared by the three groups, while 1568, 701 and 580 ASVs were the unique ASVs of the control, abscess, and surgery groups, respectively.

**Figure 3:**
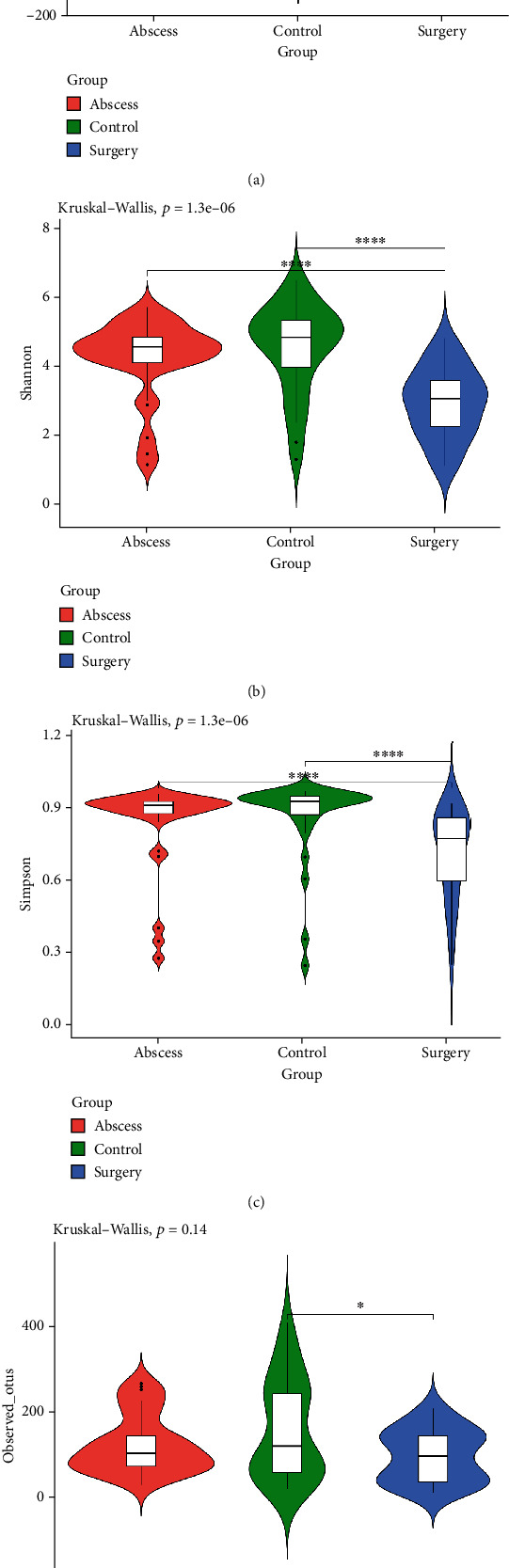
(a) Alpha diversity of gut microbiota diversity estimated by (a) Chao1 index, (b) Shannon index, (c) Simpson index, and (d) observed species index. The *p* values of the overall differences among the groups were obtained using the Kruskal-Wallis nonparametric test, and the significance level of the differences was obtained using Dunn's test after pairwise comparison between the groups (^∗^*p* < 0.05, ^∗∗^*p* < 0.01, ^∗∗∗^*p* < 0.001), and ^∗∗∗∗^*p* < 0.0001).

The abundance and uniformity of gut microbiota in each group were evaluated using the four *α*-diversity indices, including Chao1, Shannon, Simpson, and observed species indices. As shown in Figure 3, in comparison with the surgery group, the Shannon (*p* < 0.001) and Simpson (*p* < 0.001) indices of the control and abscess groups significantly decreased. Meanwhile, the observed species in the surgery groups changed significantly as compared to the control group. This indicated that the abundance and uniformity of gut microbiota altered after surgical treatment.

### 3.3. Differences in the *β*-Diversity of Gut Microbiota before and after Surgeries in the Healthy Individuals and Patients with Perianal Abscess

**Figure 4:**
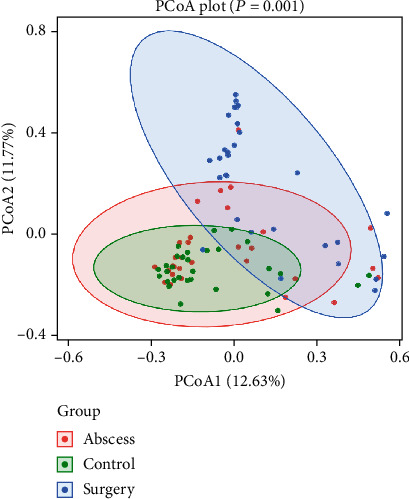
Principal coordinate analysis (PCoA) of the gut microbiota based on Bray-Curtis distance.

The PCoA based on Bray-Curtis distance was used to evaluate the *β*-diversity of the samples. As shown in Figure 4, the distance between the samples is represented by the two principal coordinates (PC1 and PC2), where the samples that are located close together were more similar in their compositions. The *p* values in Figure 4 were obtained using the Adonis test and show that the differences between the groups were significant.

### 3.4. Differences in the Abundance of Gut Microbiota between the Healthy Individuals and Patients with Perianal Abscess before and after Surgery

**Figure 5:**
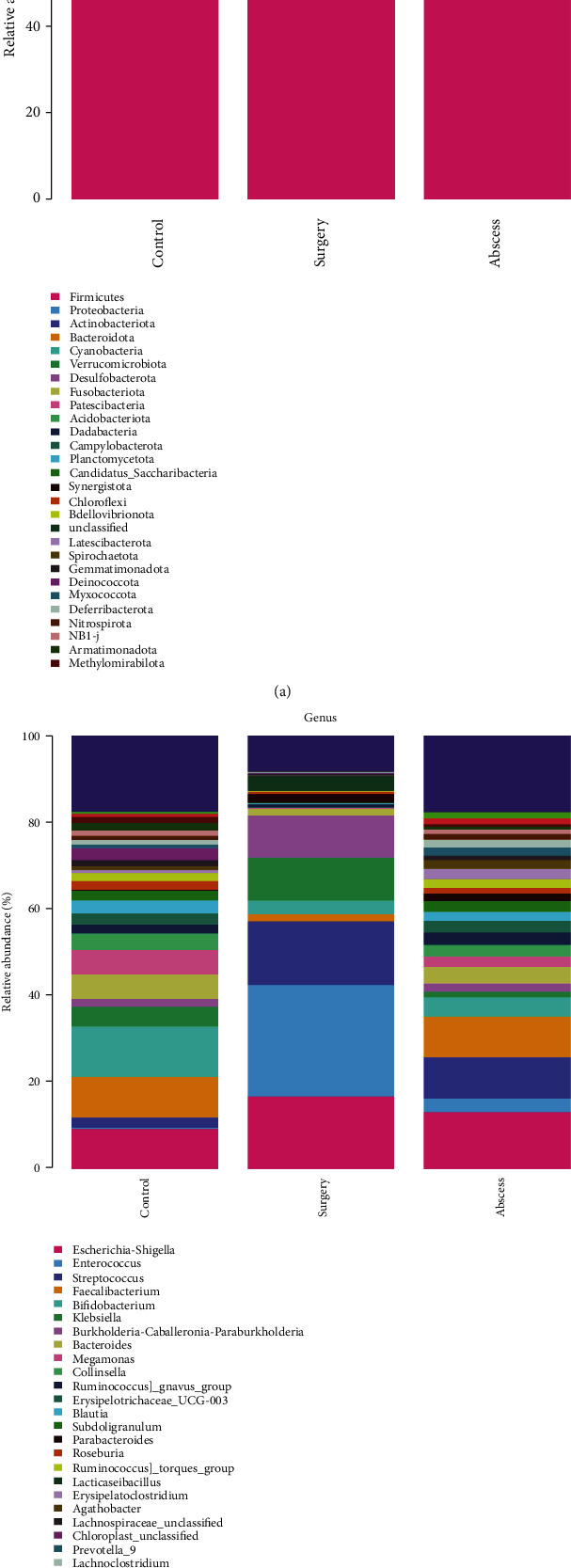
Relative abundance of bacteria at (a) phylum level and (b) genus level.

**Figure 6:**
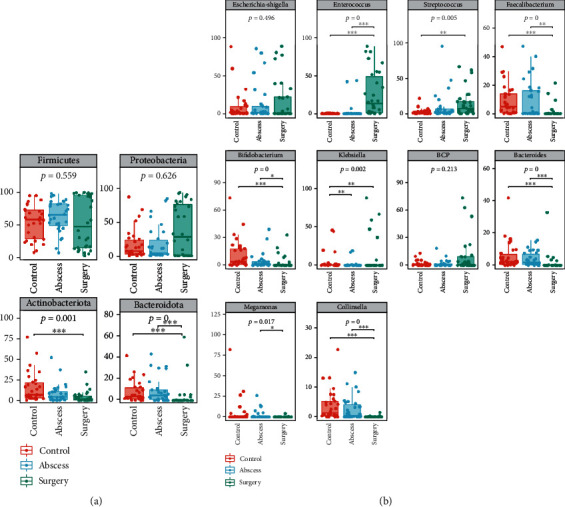
Statistical analysis of the differences in the relative abundances of bacteria at (a) phylum level and (b) genus level. The *p* values of the overall differences among the groups were obtained using the Kruskal-Wallis nonparametric test, and the significance level of the differences was obtained using Dunn's test after the pairwise comparisons between the groups (^∗^*p* < 0.05, ^∗∗^*p* < 0.01, and ^∗∗∗^*p* < 0.001).

The taxonomic compositional analysis of the gut microbiota suggested that the phyla *Firmicutes*, *Proteobacteria*, *Actinobacteria*, and *Bacteroidetes* were the main bacterial species in the intestine at phylum level (Figure 5(a)). The abundance of these gut microbiota went through significant changes (Figure 6(a)). As compared to the control group, the relative abundances of *Actinobacteriota* and *Bacteroidota* were significantly lower in the surgery group. At the same time, the relative abundance of *Bacteroidota* in the surgery group was significantly lower than that in the abscess group.

Figure 5(b) shows the 30 most abundant bacteria in the gut microbiota at the genus level. The relative abundances of these gut microbiota altered significantly among the control, abscess, and surgery groups. The top 10 most abundant bacterial species were selected for the statistical analysis of differences (Figure 6(b)). As compared to the control group, the relative abundances of *Enterococcus*, *Streptococcus*, and *Klebsiella* in the surgery group significantly increased, while those of *Faecalibacterium*, *Bifidobacterium*, *Bacteroides*, and *Collinsella* significantly decreased. Meanwhile, as compared to the surgery group, the relative abundance of *Enterococcus* in the surgery group significantly increased, while those of *Faecalibacterium*, *Bifidobacterium*, *Bacteroides*, *Megamonas*, and *Collinsella* significantly decreased.

### 3.5. Linear Discriminant Analysis Effect Size (LEfSe) Analysis of the Composition of Gut Microbiota in the Three Groups

**Figure 7:**
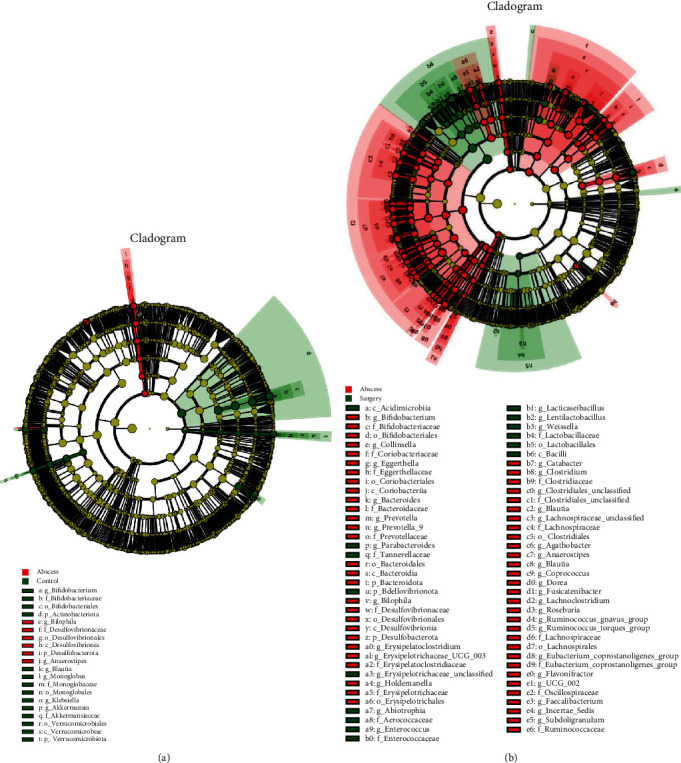
Linear discriminant analysis effect size (LEfSe) analysis of the differences in the composition of gut microbiota composition between (a) control and abscess groups and (b) abscess and surgery groups. The different circle layers show evolutionary relationships among the six taxonomic levels of phylum, class, order, family, genus, and species from the inside to the outside. The larger nodes of each level represent higher abundance of the species. The yellow and red nodes indicate the insignificant and significant differences of the species from the control group. The gates with significant differences are directly marked in the figure, and the species nodes with other different levels are marked with letters, which specifically represent species.

In order to further explore the differences in the four groups of intestinal microbial communities, LEfSe analysis was performed, which showed that the linear discriminant analysis (LDA) scores were higher than 2.0. As shown in Figures 7(a) and 7(b), the LEfSe analysis showed a significant difference between the control and abscess groups and the abscess and surgery groups. These differences should be focused on in the future.

### 3.6. Random Forest Analysis

**Figure 8:**
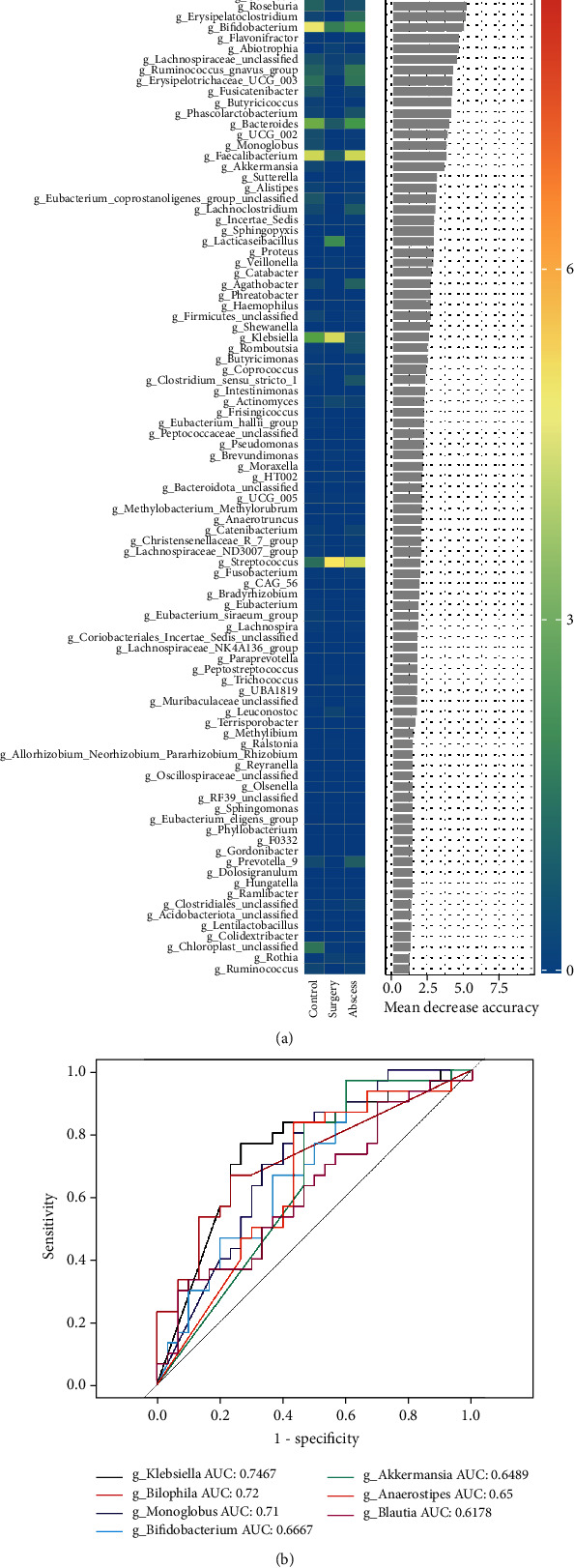
(a) Random forest analysis at genus level. (b) ROC curve analysis of the 10 different bacterial groups at genus level.

The random forest analysis at the genus level (Figure 8(a)) shows the difference contribution degree of microorganisms at the genus level among the control group, the abscess group and the surgery group. As shown in Figure 8(a), the heat map in the middle shows the change of microbial abundance at the TOP 100 genus level. It can be seen from the mean decrease accuracy on the right that seven microorganisms, *Blautia*, *Anaerostipes*, *Akkermansia*, *Bifidobacterium*, *Monolobus*, *Klebsiella*, and *Bilophila*, also showed high differential contribution.

### 3.7. Screening of Biomarkers for Perianal Abscess

Using the LEfSe analysis for the control and abscess groups, a total of 10 different bacterial groups were screened at genus level as biomarkers for perianal abscess. At the same time, the sensitivity and accuracy of the 10 different bacterial groups were verified using ROC curve (Figure 8). The ROC curve determines the sensitivity of biomarkers through area under the curve (AUC). The results of the ROC curve showed that the AUCs of *Klebsiella* and *Bilophila* were 0.7467 and 0.72, respectively, among the seven genera and could be used as potential diagnostic markers for perianal abscesses.

### 3.8. Prediction of the Function of Gut Microbiota

**Figure 9:**
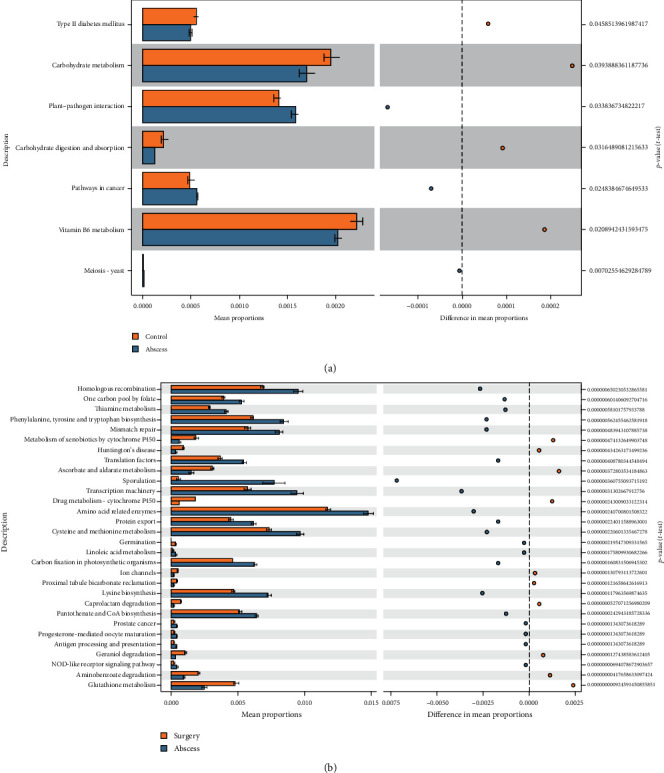
PICRUSt2 functional comparisons between the (a) control and abscess groups and (b) abscess and surgery groups. The *t*-test was used to generate this figure with a threshold value of *p* < 0.05. The results showed that the KEGG pathways were statistically significantly different in the different groups (95% CI).

The differences in the functions of gut microbiota between the control and abscess groups and abscess and surgery groups were analyzed using the PICRUSt2 software. As compared to the control group, the pathways, such as carbohydrate metabolism, plant-pathogen interaction, carbohydrate digestion and absorption, and vitamin B6 metabolism, were significantly enriched in the gut microbiota of the abscess group, which were closely related to the pathogenesis of perianal abscess (Figure 9(a)). In addition, after surgical treatment, as compared to the abscess group, the functions of gut microbiota in the surgery group were significantly enriched in thiamine metabolism, metabolism of xenobiology by cytochrome P450, ascorbate and aldate metabolism, drug metabolism-cytochrome P450, amino acid-related enzymes, lysine biosynthesis, aminobenzoate degradation, glutathione metabolism, and other pathways related to nutrient and drug metabolism (Figure 9(b)).

## 4. Discussion

In this study, the gut microbiota of the healthy people and patients with perianal abscesses were studied before and after surgeries. Previous studies have shown that the gut microbiota plays an important role in intestinal diseases, such as enteritis and colon cancer, and is closely related to the physiological metabolism, inflammation, and immune response of the intestine [14, 26, 27]. Importantly, it was found in previous studies that the intestinal diseases are often accompanied by changes in the diversity and abundance of gut microbiota, indicating that each microorganism or the entire gut microbiota are the cause of intestinal diseases [13–15]. Anus is the gateway of digestive tract to the outside of body. Perianal abscesses might be related to gut microbiota. The studies on the microbiota around the anus are limited, and the data on the microbial abundance and diversity is not enough. Therefore, evaluating the abundance of microorganisms and determining their types, clarifying the role of gut microbiota in the occurrence and development of perianal abscesses, and enabling individualized treatment for the patients of perianal abscesses similar to cancer precision medicine are highly important.

In this study, significant changes in interleukin 6 and tumor necrosis factor-*α* in the blood of healthy subjects, patients with perianal abscesses, and patients after perianal abscess surgery were analyzed. The *16S rRNA* gene sequencing results showed the changes in the gut microbiota between the healthy individuals and patients with perianal abscesses before and after surgeries. Venn diagrams and *α*-diversities indicated the differences in the abundance and uniformity of gut microbiota between the healthy individuals and patients with perianal abscesses before and after surgeries. In addition, *β*-diversity indicated that the grouping effects among the three groups were good. The analysis of the classification and composition of gut microbiota showed significant differences in that of healthy individuals and patients with perianal abscesses before and after surgeries. In addition, LEfSe analysis was used to compare the differences in the gut microbiota between healthy individuals and patients with perianal abscesses before and after surgeries. These differential gut microbiota at the genus level screened by the LEfSe analysis might be used as diagnostic markers for perianal abscess. Since the treatment effects on the patients with perianal abscess before and after surgeries were not evaluated, the gut microbiota that can evaluate the prognosis of patients with perianal abscess for ROC curve analysis were not screened. However, the differential microbiota in the patients with perianal abscesses before and after surgeries were selected using the LEfSe analysis, which might provide a reference for the postoperative treatment and medication of the patients with perianal abscesses.

In this study, a total of 7 gut microbial species were validated, which could differentiate the healthy individuals from the patients with perianal abscesses. The results of the random forest analysis and ROC curve analysis showed that the classification effects of *Klebsiella* and *Bilophila* were better. *Klebsiella* is a parasite of the respiratory tract or intestinal tract of animals and an opportunistic pathogen. It can cause human pneumonia and nosocomial bacterial infection [28]. A diversity study of *Klebsiella oxytoca* and related bacteria based on the publicly available shotgun metagenomic datasets suggested that approximately one in 10 neonatal stool samples contained *Klebsiella* spp. [29]. Another study showed that the high-alcohol-producing *Klebsiella pneumoniae* was closely related to alcoholic fatty liver disease [30]. All these studies demonstrated the potential of using *Klebsiella* as a diagnostic marker for disease. *Bilophila*, as a sulfur-producing bacterium in the intestine, is also closely related to other intestinal diseases [31]. Studies have shown that some of the sulfur-producing bacteria, such as *Fusobacterium*, *Desulfovibrio*, and *Bilophila wadsworthia*, affect the progression of colorectal cancer by producing hydrogen sulfide [32]. In addition, other studies have shown that *Bilophila wadsworthia*, which grows in foods containing red-processed meat proteins, can erode the mucus layer on the surface of colon, thereby allowing more bacterial flora to approach the lining cells and thus promote inflammation. Perianal abscess is closely related to inflammation [33]. Therefore, *Bilophila* might be used as a potential biomarker for the diagnosis of perianal abscess.

In the postoperative treatment of perianal abscesses, some pathogenic bacteria such as *Escherichia coli*, *Bacteroides*, *Streptococcus* and *Staphylococcus*, and other acquired resistant strains are frequently detected [34]. Therefore, it is particularly important to monitor the pathogenic organisms that may be present in the postoperative perianal abscess. In our results, the three microorganisms *Enterococcus*, *Streptococcus*, and *Bacteroides* in the genus level after surgery for perianal abscess were dominant and differed significantly. *Enterococcus cecorum*, also a common hospital infection, was first found in the cecum of chickens [35]. *Enterococcus* was significantly elevated in the surgery group compared to the abscess group, suggesting that we should pay extra attention to the effects of *Enterococcus* after surgery for perianal abscess. The clinically isolated *β-hemolytic streptococci* and *Streptococcus pyogenes* of the genus *Streptococcus* are also common pathogenic bacteria that cause purulent infections [36]. In the results, *Streptococcus* was significantly higher in the surgery group compared to control. Therefore, we should also pay extra attention to *Streptococcus* in the postoperative treatment of perianal abscesses. Finally, *Bacteroides* decreased significantly in the surgery group compared to the abscess group. The effects of *Bacteroides* on the body are bidirectional [35]. Some beneficial *Bacteroides* are involved in the metabolic regulation of the body, but *B. fragilis* is the most common and often isolated from clinical specimens and is considered the most virulent *Bacteroides* [35]. Due to limitations in 16S rRNA gene sequencing, we were unable to accurately distinguish harmful bacteria in the genus *Bacteroides*. Therefore, it is not possible to determine the effects of harmful bacteria of the genus *Streptococcus* for the time being. These findings will guide postoperative antibiotic therapy for perianal abscesses.

The differences in the function of gut microbiota before and after surgery in the healthy individuals and patients with perianal abscesses before and after surgeries were compared. As compared to the control group, the plant-pathogen interaction pathway was significantly enriched in the abscess group, which suggested that some pathogenic bacteria might be colonized in the intestines of the patients with perianal abscesses. In addition, a significant decrease in the abundance of gut microbiota enriched in metabolism-related pathways, such as carbohydrate metabolism, carbohydrate digestion, and absorption, and vitamin B6 metabolism indicated that the patients with perianal abscess might have a metabolic disorder, which was consistent with the results of a previous intestinal disease study [12].

At the same time, the functions of the different gut microbiota in the patients with perianal abscesses before and after surgery were also compared. Among them, as compared to the abscess group, the gut microbiota in the surgery group were significantly enriched in the drug metabolism-cytochrome P450 pathway. This result indicated that, after surgery, intestinal drug metabolism in the patients with perianal abscess might increase. This might also be related to the patient's medication after surgery. It was worth noting that, as compared to the abscess group, the glutathione metabolism pathway in the surgery group was also significantly enriched. Glutathione helps in maintaining the normal functioning of immune system and has antioxidant and detoxifying effects. The changes in the concentration of glutathione in the intestines of patients with perianal abscess might be accompanied by the effects on the intestinal immune function and oxidative stress, which require further verification in future studies [37, 38]. In addition, the gut microbiota was differentially enriched in thiamine metabolism, metabolism of xenobiology by cytochrome P450, ascorbate and aldehyde metabolism, amino acid-related enzymes, lysine biosynthesis, and aminobenzoate degradation pathways, which also indicated the disorder of amino acid and thiamine metabolism in the patients with perianal abscess after surgery [39–41].

This study screened the differences in the gut microbiota of healthy individuals and patients with perianal abscess before and after surgeries and also screened the biomarker *Bilophila* for perianal abscess. However, more detailed aspects of these findings should be clarified in future studies, such as studying the effects of fecal transplantation or antibiotic intervention, in order to better characterize the differential gut microbiota and verify the biomarker *Bilophila* selected in this study.

## Data Availability

The data used to support the findings of this study are available from the corresponding author upon request.
